# Diagnostic Accuracy of Infection Markers to Diagnose Infections in Neonates and Children Receiving Extracorporeal Membrane Oxygenation

**DOI:** 10.3389/fped.2021.824552

**Published:** 2022-01-26

**Authors:** Irene Doo, Lukas P. Staub, Adrian Mattke, Emma Haisz, Anna Lene Seidler, Nelson Alphonso, Luregn J. Schlapbach

**Affiliations:** ^1^Paediatric Intensive Care Unit, Queensland Children's Hospital, Brisbane, QLD, Australia; ^2^Child Health Research Centre, The University of Queensland, Brisbane, QLD, Australia; ^3^National Health and Medical Research Council (NHMRC) Clinical Trials Centre, University of Sydney, Sydney, NSW, Australia; ^4^Queensland Paediatric Cardiac Service, Queensland Children's Hospital, Brisbane, QLD, Australia; ^5^Department of Intensive Care Medicine and Neonatology, and Children's Research Center, University Children's Hospital of Zurich, University of Zurich, Zurich, Switzerland

**Keywords:** C-reactive protein, extracorporeal membrane oxygenation, extracorporeal life support, infection, pediatric, procalcitonin, white cell count, diagnostic accuracy

## Abstract

**Background:**

Infections represent one of the most common complications in patients managed on Extracorporeal Membrane Oxygenation (ECMO) and are associated with poorer outcomes. Clinical signs of infection in patients on ECMO are non-specific. We assessed the diagnostic accuracy of Procalcitonin (PCT), C-reactive protein (CRP) and White cell count (WCC) to diagnose infection on ECMO.

**Methods:**

Retrospective single center observational study including neonates and children <18 years treated with ECMO in 2015 and 2016. Daily data on PCT, CRP and WCC were assessed in relation to microbiologically confirmed, and clinically suspected infection on ECMO using operating characteristics (ROC) curves.

**Results:**

Sixty-five ECMO runs in 58 patients were assessed. CRP had the best accuracy with an area under the ROC curve (AUC) of 0.79 (95%-CI 0.66–0.92) to diagnose confirmed infection and an AUC of 0.72 (0.61–0.84) to diagnose confirmed and suspected infection. Abnormal WCC performed slightly worse with an AUC of 0.70 (0.59–0.81) for confirmed and AUC of 0.66 (0.57–0.75) for confirmed and suspected infections. PCT was non-discriminatory.

**Conclusion:**

The diagnosis of infections acquired during ECMO remains challenging. Larger prospective studies are needed that also include novel infection markers to improve recognition of infection in patients on ECMO.

## Highlights

- When comparing C-reactive protein (CRP), white cell count (WCC), and procalcitonin (PCT) to diagnose infection in 65 neonatal and pediatric ECMO runs, the performance of CRP was best.
- Combining infection markers did not lead to improved performance to diagnose sepsis, and PCT was non-discriminatory.
- This study can inform the design of prospective multicenter studies on novel infection markers and marker algorithms to improve recognition of infection in patients on ECMO.

## Introduction

Extracorporeal Membrane Oxygenation (ECMO) has gained widespread acceptance as rescue treatment for refractory cardiovascular and respiratory failure in adults and children (1–4). The use of ECMO has extended to increasingly complex patient populations, such as patients with malignancies (5, 6) and patients with chronic medical comorbidities or those requiring extracorporeal cardiopulmonary resuscitation (ECPR) (7, 8). This complex patient group is subject to high risk of developing complications and more likely to be colonized with multi-drug resistant organisms (9).

Infections represent one of the most common complications in patients managed on ECMO (10). A recent review of the literature revealed an incidence of ECMO-associated infections between 10.1 and 116.2 per 1,000 ECMO days (11). Nosocomial infections acquired on ECMO have been described in up to 64% of adult and 30% of neonatal ECMO runs and are associated with increased ECMO duration, prolonged mechanical ventilation, prolonged hospital stay, and increased mortality; particularly in presence of invasive fungal infections (12).

Clinical signs of infection in patients on ECMO are non-specific and often masked, making a diagnosis of infection challenging (10). During ECMO, respiratory rate and temperature are usually artificially controlled, and ECMO perfusion, sedation, and cardiovascular drugs alter heart rate and blood pressure. In addition, ECMO-triggered systemic inflammation can mimic features of sepsis. A definite diagnosis of infection therefore relies on microbiological cultures which commonly take up to 48–72 h to analyse (13). Blood culture performance depends on bacterial load, and results can be falsely negative in patients exposed to prophylactic or therapeutic antibiotics (14). This diagnostic uncertainty may lead to unnecessary treatment with antibiotics and may promote antibiotic resistance (15). At the same time, missing a diagnosis of a nosocomial infection will delay initiation of effective antimicrobial treatment and potentially contribute to excess ECMO mortality and morbidity (16, 17).

Laboratory markers of infection have gained widespread acceptance to guide decisions to start and stop antibiotics in critical care (18). Extracorporeal circuits and shock-related acute phase responses lead to non-specific increases in infection markers, further confounding the diagnosis of infection in this population (19). In addition to the traditional infection markers White Cell Count (WCC) and C-reactive protein (CRP), a large randomized-controlled trial in adult general ICU patients demonstrated superiority of a Procalcitonin (PCT) guided approach (20). Similarly, PCT guided therapy in neonates with suspected early-onset sepsis allowed to reduce the duration of antimicrobial therapy (21, 22). While observational studies in critically ill children suggest superiority of PCR over CRP, adequately powered RCTs are lacking (23). In contrast, the available evidence supporting the use of infection markers on ECMO is very limited: Only four studies across 65 adult and 47 neonatal/pediatric patients reported on the accuracy of markers to diagnose infection in patients already on ECMO (19, 24–26).

We hypothesized that PCT would be superior to CRP and WCC to diagnose infection in neonates and children treated with ECMO. For this purpose, we assessed the diagnostic accuracy of daily measured PCT, CRP, and WCC to diagnose infection in 65 consecutive ECMO runs at our center.

## Methods

We performed a retrospective, single center cohort study of all consecutive neonatal and pediatric ECMO runs at the Queensland Children's Hospital Pediatric Intensive Care Unit (PICU). Ethics approval was obtained by the institutional human research and ethics committee including waiver of consent (HREC/17/QRCH/8). This study was reported following the current Standards for Reporting Diagnostic accuracy studies (STARD) guideline (27).

We included ECMO cases in neonates, children and adolescents aged <18 years with initiation of ECMO between 01/01/2015 and 31/12/2016. All types of runs were included [venovenous (VV), and venoarterial (VA), extracorporeal cardiopulmonary resuscitation (ECPR), cardiac, respiratory]. Two authors independently reviewed the institutional patient data management system (Metavision, iMDsoft, USA) of all patients treated with ECMO during the study period to ascertain which patients had a microbiologically proven infection, suspected infection or no infection on each ECMO day.

The ECMO service provides centralized ECMO support for the state of Queensland and northern New South Wales including ECPR, cardiac, and respiratory ECMO for all neonatal and pediatric age groups. The default ECMO setup included centrifugal pumps, a shunt containing a filter for slow continuous ultrafiltration, in- and outlet pressure monitors, and continuous measurement of venous and arterial saturations, hemoglobin, and ECMO flow. Steroids are not routinely used. Antimicrobial prophyaxis using cephazolin is used for the first 24 h after cannulation, and in children with open chest for the duration of the chest remaining open.

### Definitions

Microbiologically confirmed infection was defined as per Center for Diseases Control guideline definitions, characterized by positive bacterial or fungal growth of microbiological samples obtained in blood, urine, or endotracheal aspirate (28), and treated with intravenous antibiotics for at least 5 days or until death. Clinically suspected infection was defined as suspicion of infection documented in clinical notes in presence of negative cultures and treated with intravenous antibiotics for at least 5 days or until death.

We searched the electronic health records, radiology results and laboratory data for information on the focus of infection, to categorize them into Bloodstream Infection (BSI); Central Line Associated Blood Stream Infection (CLABSI); urinary tract infection (UTI); and Ventilator Associated Pneumonia (VAP). VAP was defined as clinical and radiological signs consistent with acute respiratory infection in presence of likely pathogenic microbiological organisms in sputum (10). Positive endotracheal cultures in the absence of clinical or radiological signs were considered as colonization. Patients with confirmed viral infections and patients who received prophylactic antibiotics in the absence of suspected or confirmed infections were considered non-infected. In episodes of confirmed infection, we defined day 0 as the day when a culture was obtained that later confirmed infection, while in episodes of suspected infection it was the day when intravenous antibiotics were started. We considered ECMO days from 48 h prior to day 0 until the end of antibiotic treatment as infected days (confirmed or suspected). Days outside this time frame, and days in ECMO runs where no infection was suspected or confirmed, were considered not infected.

During the study period, institutional practice as per the local ECMO guideline recommended obtaining daily surveillance cultures, PCT, CRP, and WCC. For the purpose of this study, daily laboratory and microbiology data (highest daily PCT, CRP, WCC; as well as microbiological cultures from blood, urine and endotracheal aspirate) were reviewed from the institutional ICU database from the day prior to ECMO to day of decannulation or death. Changes in CRP and PCT over 48 h were calculated to assess whether changes in biomarkers were more accurate in detecting infection than absolute values. Patient and ECMO characteristics including age, severity on presentation, surgical procedures performed, circuit changes, renal replacement therapy, inotrope use, daily maximum ECMO flow rates, PICU and hospital length of stay, and mortality, were extracted from the prospective institutional ECMO and ICU databases.

### Statistical Analysis

We divided the ECMO runs into three groups according to the occurrence of infections: (1) patients with no infection during the whole ECMO run, (2) runs with a suspected infection, (3) runs with a confirmed infection. If a patient had both confirmed and suspected infection episodes during a run, this particular run was grouped under confirmed infection. Median and interquartile ranges (IQR) were used to describe continuous data. Given the small sample size and uncertain normality of data, non-parametric tests were used for group comparisons (Kruskal-Wallis test and Spearman Rank correlation for continuous data, Fisher's Exact test for proportions).

We used the biomarker measurements obtained on day 0 of each episode to estimate their accuracy in predicting a bacterial or fungal infection. If markers were not obtained at the same time as blood cultures, we used the highest marker value available within no more than 24 h of blood culture sampling. Given that both very low and very high WCC can be indicative of an infection, we applied age-dependent categorization of WCC based on the 2005 International Pediatric Sepsis Definition Consensus Conference (29).

We used receiver operating characteristics (ROC) analyses to compare the accuracy of markers by calculating the area under the ROC curve (AUC). As repeated measurements within ECMO runs may be correlated, the estimation of 95% confidence bands around ROC curves was adjusted for clustered data. For PCT and CPR, we determined the test cut-off values resulting in 80 and 90% sensitivity, respectively, which may serve as minimal thresholds for clinical practice. For completeness, we also used Youden's Index to find marker cut-offs yielding maximum combined sensitivity and specificity (30, 31). For each test cut-off, we calculated sensitivity, specificity, positive likelihood ratio (LR+), negative likelihood ratio (LR-), positive predictive value (PPV) and negative predictive value (NPV).

We further tested whether combining the two best performing markers would increase the diagnostic accuracy. In exploratory analyses, we tested for associations between infection markers and ECMO parameters, including ECMO type, patient age (dichotomized into neonates vs. older children), day of ECMO, peak daily lactate, and peak daily flow rate. We built multi-level regression models to assess the association of ECMO parameters with infection markers, adjusting for repeated measurements within ECMO runs.

All analyses used pairwise deletion, and no imputation of missing values was performed. A *p*-value < 0.05 was considered significant. Open source software R (32) (in particular pROC and clusteredROC packages), and SAS version 9.4 were used for analyses.

## Results

### Characteristics of Study Cohort

During the study period, 65 ECMO runs with an average run duration of 7.2 days (median 5 days, IQR 2–9) were performed in 58 patients. The cohort included 32 (49%) neonatal, 33 (51%) pediatric ECMO runs of which 12 (18%) were ECPR, 22 (34%) cardiac, and 31 (48%) respiratory, respectively (Table 1). Of these, 31 (48%) runs were with no infection, 13 (20%) with suspected infection, and 21 (32%) with confirmed infection (Table 1). In 20 of the 65 runs the patients died, resulting in a mortality of 31%.

**Table 1:** Baseline characteristic of patients, grouped into Extracorporeal Membrane Oxygenation (ECMO) runs with no infection, with suspected infection, and with confirmed infection.

	**Runs with no infection, *N* = 31 (48%)**	**Runs with suspected infection, *N* = 13 (20%)**	**Runs with confirmed infection, *N* = 21 (32%)**	
**Characteristic**	**Median (IQR) or N (%)**	**Median (IQR) or N (%)**	**Median (IQR) or N (%)**	***p*-value**
**Demographics**				
Age in days	15 (4–230)	14 (1–138)	141 (32–1,327)	0.06
Sex (male)	19 (61)	5 (38)	12 (57)	0.40
Weight (kg)	3.5 (3.0–4.4)	3.6 (3.0–4.7)	5.2 (4.2–20.0)	0.02
**ECMO type**				
Neonatal	18 (58)	9 (69)	5 (24)	0.01
Pediatric	13 (42)	4 (31)	16 (76)	
VV	3 (10)	3 (23)	6 (29)	0.17
VA	28 (90)	10 (77)	15 (71)	
Respiratory	9 (29)	8 (62)	14 (67)	0.03
Cardiac	15 (51)	2 (15)	4 (19)	
ECPR	6 (19)	3 (23)	3 (14)	
Run duration (hrs)	95 (46–144)	166 (56–237)	177 (70–360)	0.06
Survival	24 (77)	9 (69)	12 (57)	0.28
ICU LOS (hrs)	334 (144–811)	288 (105–526)	1,024 (256–1,652)	0.06
Hospital LOS (hrs)	971 (344–2,947)	401 (271–928)	2,154 (525–2,514)	0.14
**Severity pre ECMO**				
PIM3	0.067 (0.015–0.208)	0.082 (0.042–0.131)	0.058 (0.012–0.082)	0.25
Pre ECMO pH	7.22 (7.03–7.32)	7.26 (7.15–7.36)	7.26 (7.19–7.35)	0.42
Pre ECMO pCO_2_	53 (44–74)	46 (34–54)	49 (41–57)	0.19
Pre-ECMO pO_2_	44 (28–97)	55 (41–88)	63 (48–84)	0.27
Pre ECMO SaO_2_	76 (51–97)	89 (76–97)	93 (82–97)	0.45
Pre-ECMO HCO_3_	21 (16–24)	21 (16–23)	22 (18–26)	0.61
4 Hr patient flow	0.48 (0.40–0.67)	0.46 (0.40–0.66)	0.54 (0.41–1.46)	0.55
24 Hr patient flow	0.45 (0.33–0.59)	0.44 (0.37–0.68)	0.52 (0.41–1.13)	0.27

*If a patient had both confirmed and suspected infection episodes during a run, the run was grouped under confirmed infection*.

*ECMO, Extracorporeal Membrane Oxygenation; ECPR, Extracorporeal Cardiopulmonary Resuscitation; LOS, length of stay; VA, venoarterial; VV, venovenous*.

Out of 590 ECMO days, antibiotics were administered on 512 days (87%), including 155 days for prophylaxis, 122 days for suspected and 182 days for confirmed infection, and 53 days to treat a necrotizing enterocolitis. Surveillance blood cultures were taken on 492 run days (86%) out of 575 days where this information was available, with 17 (3.5%) blood cultures resulting positive. The most commonly identified organisms were *Candida* spp. and *Stenotrophomonas* spp., followed by *Enterobacter* spp., Coagulase-negative staphylococci (CoNS), and *Klebsiella* spp. The most common focus of infection was VAP, followed by UTI (Supplementary Table A). PCT was available for 252 days (43%), with a median of 5.8 mg/L (IQR 1.6–14.5 mg/L); CRP was available for 404 ECMO days (68%), median 63 mg/L (IQR 24–131 mg/L); and WCC was available for 557 ECMO days (94%), median 12.2^*^10^9^/L (IQR 8.6–17.6^*^10^9^/L).

### Diagnostic Accuracy of Infection Markers

**Figure 1:**
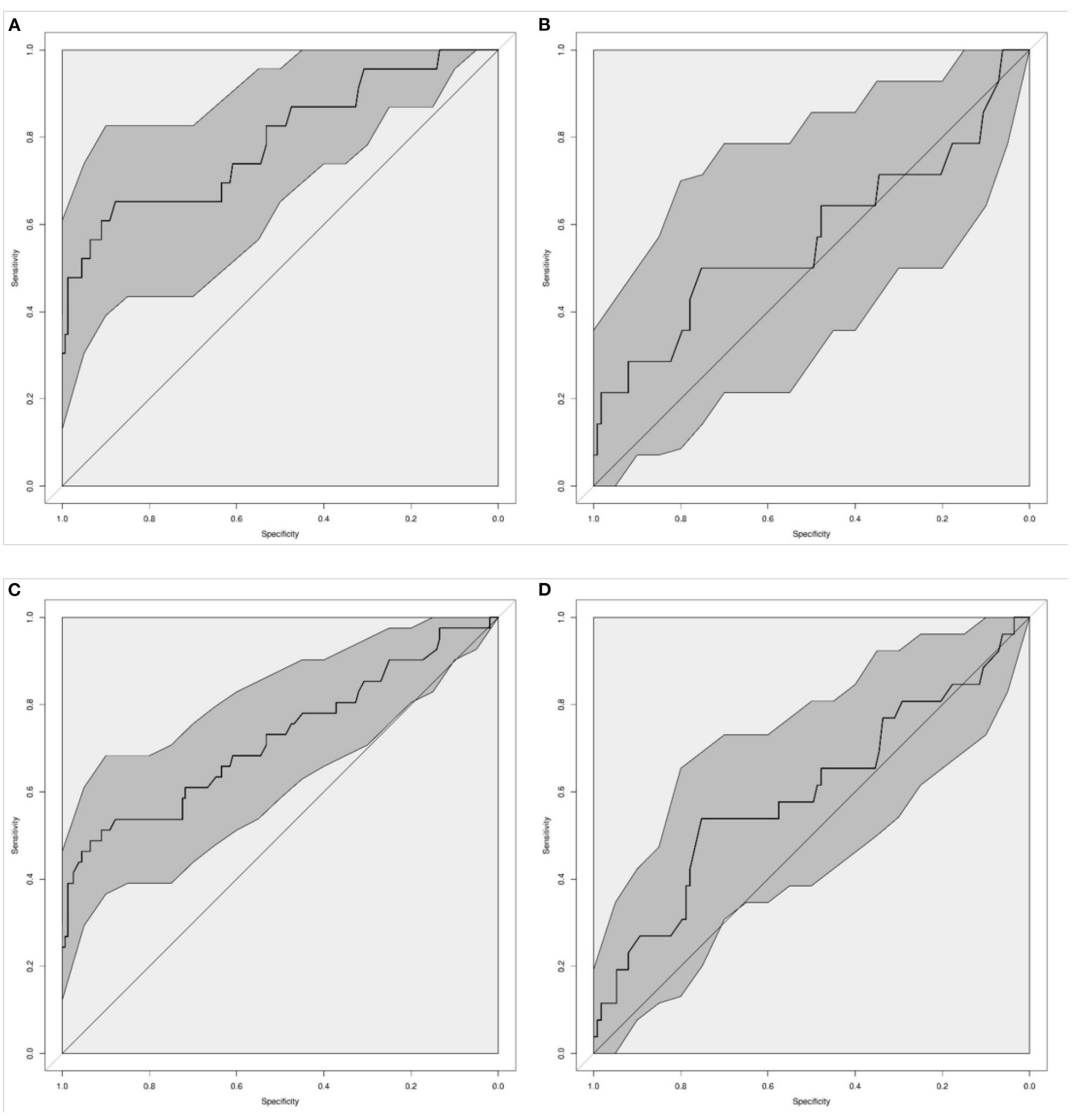
Diagnostic accuracy of C-Reactive Protein [CRP; **(A,C)**] and Procalcitonin [PCT; **(B,D)**] to diagnose infection in neonates and children on ECMO. Graphs show the Receiver-Operating Characteristic Area Under the Curve (AUC) for confirmed infection **(A,B)**, and for confirmed and suspected infection **(C,D)**. Dark gray shaded areas indicate 95%-Confidence bands.

The diagnostic accuracy to identify confirmed infection was best for CRP with an AUC of 0.79 (95% CI 0.66–0.92), and it was worst for PCT with an AUC of 0.57 (95% CI 0.35–0.80, Table 2, Figures 1A,B) and. The accuracy of WCC was intermediate (AUC 0.70, 95% CI 0.59–0.81, Supplementary Figure 1). A change in PCT over 48 h was not significantly associated with an infection (AUC 0.68, 95% CI 0.49–0.86), neither was an increase in CRP over 48 h (AUC 0.65, 95% CI 0.47–0.84).

**Table 2:** Diagnostic accuracy of Procalcitonin (PCT), C-reactive protein (CRP), and White Cell Count (WCC) to diagnose infection on ECMO.

**(A) Confirmed infections**									
**Marker**	**Model**	**Cut-off**	**ROC AUC (95% CI)**	**Se**	**Sp**	**LR+**	**LR-**	**PPV**	**NPV**
PCT			0.573 (0.351, 0.795)						
	Se 0.8	0.7 mg/L		0.80	0.11	0.90	1.82	0.10	0.82
	Se 0.9	0.6 mg/L		0.90	0.08	0.98	1.25	0.11	0.87
	Youden^**^	11.5 mg/L	0.50	0.75	2.00	0.67	0.20	0.92	
CRP			0.792 (0.662, 0.922)						
	Se 0.8	41.5 mg/L		0.80	0.53	1.70	0.38	0.20	0.95
	Se 0.9	21.8 mg/L		0.90	0.32	1.32	0.31	0.16	0.96
	Youden^**^	102.5 mg/L	0.65	0.88	5.42	0.40	0.44	0.94	
WCC	WCC abnormal^*^	0.703 (0.591, 0.814)							
				0.65	0.75	2.60	0.47	0.22	0.95
WCC or CRP	WCC abnormal^*^ or CRP>41.5 mg/L		0.640 (0.532, 0.749)						
				0.77	0.51	1.57	0.45	0.15	0.95
**(B) Confirmed and suspected infections**									
**Marker**	**Model**	**Cut-off**	**ROC AUC (95% CI)**	**Se**	**Sp**	**LR+**	**LR-**	**PPV**	**NPV**
PCT			0.597 (0.430, 0.764)						
	Se 0.8	1.9 mg/L		0.80	0.30	1.14	0.67	0.21	0.87
	Se 0.9	0.6 mg/L		0.90	0.09	0.99	1.11	0.19	0.80
	Youden^**^	11.5 mg/L	0.54	0.75	2.16	0.61	0.33	0.88	
CRP			0.722 (0.606, 0.839)						
	Se 0.8	25.5 mg/L		0.80	0.37	1.27	0.54	0.24	0.91
	Se 0.9	16.5 mg/L		0.90	0.25	1.20	0.40	0.24	0.91
	Youden^**^	124.0 mg/L	0.49	0.94	8.17	0.54	0.67	0.87	
WCC	WCC abnormal^*^	0.661 (0.571, 0.752)							
				0.57	0.75	2.28	0.57	0.29	0.91
WCC or CRP	WCC abnormal^*^ or CRP>25.5 mg/L		0.607 (0.529, 0.685)						
				0.79	0.43	1.37	0.50	0.20	0.92

*Receiver Operating Characteristics Area under the Curve (ROC AUC), sensitivity (Se), specificity (Sp), positive likelihood ratio (LR+), negative likelihood ratio (LR-), positive predictive value (PPV), and negative predictive value (NPV) are shown. Results are presented for episodes of confirmed infection in comparison to non-infected controls, and episodes of confirmed or suspected infection in comparison to non-infected controls*.

* *For WCC, values were defined as upper and lower thresholds as per Goldstein et al. (29)*.

** *Cut-off according to Youden's Index*.

We then calculated cut-offs where the markers reached a sensitivity of 80 and 90%, and the cut-offs according to Youden's Index (Table 2 and Supplementary Figure 2). For CRP, cut-offs of 41.5 mg/L and 21.8 mg/L were found to diagnose a confirmed infection with 80 and 90% sensitivity, which were accompanied by specificities of 53 and 32%. Applying Youden's Index resulted in a cut-off close to 100 mg/L, which was associated with a sensitivity of 65% and a specificity of 88%. The sensitivity of abnormal WCC to diagnose proven infection was 65%, with a specificity of 75%. Combining abnormal WCC with CRP ≥41.5 mg/L resulted in worse performance than CRP alone (AUC 0.64, 95% CI 0.53–0.75).

When assessing the diagnostic accuracy to identify confirmed and suspected infection, CRP again performed best with an AUC of 0.72 (95% CI 0.61–0.84) (Figures 1C,D). A CRP of ≥25.5 mg/L diagnosed proven or suspected infection with a sensitivity of 80%, with a specificity of 37%. The sensitivity of abnormal WCC to diagnose proven or suspected infection was 57%, with a specificity of 75%. The diagnostic accuracy decreased when combining WCC and CRP (Table 2).

### Exploratory Analyses on Biomarker Associations With ECMO Run Characteristics

Using regression models, we then explored whether ECMO run characteristics were associated with biomarker levels (Supplementary Table B and Supplementary Figure 3). ECMO type and patient age were not associated with PCT, CRP or WCC. Both PCT and WCC decreased over time, with a weak but significant negative slope in the regression analysis. WCC was positively associated with peak daily lactate, while PCT and CRP increased with higher peak daily flow rates.

## Discussion

Infections are one of the most common complications for patients on ECMO and contribute to higher morbidity and mortality (12). While the benefit of rapid identification and treatment of sepsis has been broadly recognized in critically ill patients (16, 33), accurate and timely diagnosis of infections on ECMO poses a major challenge, and the evidence to guide best practice in this area remains scarce. Due to profound activation of inflammatory pathways on patients requiring ECMO and aggravated by the extracorporeal circuit itself, it is unclear to what extent widely used infection (or inflammation) markers provide clinically meaningful diagnostic accuracy in this patient group (10). We assessed the diagnostic accuracy of PCT, CRP and WCC to diagnose bacterial infections in 65 neonatal and pediatric ECMO runs. While CRP performed best, its accuracy was only moderate with an AUC below 0.8. PCT did not discriminate reliably in our cohort. Neither changes in CRP or PCT, nor combinations of biomarkers improved the performance of the diagnostic approach.

Our findings contrast with self-reported practice in a recent survey of 147 intensivists from 91 ECMO centers across 25 countries, which identified that rising CRP or PCT were the most commonly used criteria to diagnose infection (34). Our study demonstrates that patients are exposed to antibiotics on almost 90 percent of ECMO days, indicating an urgent need for improved markers or infection prediction models. Previous studies in patients on ECMO showed that WCC, absolute neutrophil count, immature to total white cell ratio, platelet count, lactate and fibrinogen were not predictive of bloodstream infections (35–38). In adult general ICU patients with sepsis and septic shock, PCT has been shown to improve the diagnosis of bacterial infections compared to traditional infection markers such as WCC or CRP, allowing to enhance antimicrobial stewardship by reducing duration of antibiotic therapy with potential mortality benefit (20, 22). A recent literature review identified 59 full text articles reporting on infections on ECMO, of which only four investigated the role of infection markers (11). These previous studies on infection markers on ECMO were very small, including <30 runs, and yielded inconclusive results (19, 24, 25, 39): Rungatscher et al. reported that neither CRP or PCT were predictive of infection in 20 children on ECMO. In contrast, a recent report on 27 neonatal and pediatric ECMO runs where the proportion of positive cultures was very high (19% BSI) observed a sensitivity and specificity of a PCT threshold of 0.5 ng/ml to diagnose infection of 92%, and 43%, respectively. In two studies on 27 and 41 adult patients, a PCT cut-off of 2 ng/ml was reported as optimal to discriminate presence of infection vs. inflammation on ECMO, however was only predictive of infection on VA ECMO but not on VV ECMO. All studies were retrospective single center studies applying different definitions of infection, they reported on one or two markers rather than comparing CRP, PCT, and WCC, and none adjusted for repeated measurements.

Our findings are concordant with a number of studies showing that WBC have poor to moderate performance to discriminate infection in children (30, 37, 40, 41). In addition, it is possible that our approach to dichotomizing WBC (in order to capture both abnormal high, as well as abnormal low WBC) may have affected results. Of note, the diagnostic accuracy to diagnose infections on ECMO decreased when CRP and WBC were combined—which may be explained by overlaying areas of poor specificity with poor sensitivity. In addition, a multitude of non-infectious factors on ECMO may affect both high and low WBC counts, such as stress, steroids, massive transfusion, and chylus loss.

In our study, PCT was elevated in more than 75% of measurements to levels above the threshold commonly applied to diagnose infection in non-ECMO patients, and was unable to discriminate between confirmed or suspected infection and no infection. However, PCR values were available only for 43% of ECMO days, potentially biasing the findings. Nonspecific PCT increase has been observed in a range of settings such as ischemia, reperfusion injury or following cardiopulmonary bypass (24, 25, 42). This likely reflects the inflammatory response to the ECMO circuit immediately following cannulation and may be further aggravated by acute organ dysfunction, shock, and reperfusion injury. Exposure to non-epithelialized extracorporeal surfaces rapidly leads to systemic inflammation as a result of profound activation of cellular and humoral components of the innate immune system (43). We therefore hypothesize that the observed trend for PCT levels to decrease over time during ECMO runs may reflect the progression of the initial inflammatory response to ECMO. While infections are more commonly acquired after the first week on ECMO, our observations suggest that different infection marker thresholds may need to be considered depending on time spent on ECMO. However, our study was not powered to define infection marker thresholds specific for each week on ECMO, and the observed inter-patient variability was very high.

This study has several limitations. Due to the retrospective design, the definition of infection relied on a combination of microbiological findings, medical notes, and the initiation of antibiotic therapy for at least 5 days or until death. As clinical decision making was not blinded to infection markers, the reference standard was not totally independent of the index tests (incorporation bias), as requested by STARD (27). Moreover, the rate of confirmed BSI was low and the majority of infection episodes were classified as VAP. This may have impaired the quality of the reference standard, as differentiation of colonization and infection is challenging in pulmonary cultures. Aligned with CDC recommendations, positive endotracheal cultures in the absence of clinical or radiological signs were considered as colonization, however we cannot rule out that some of these findings resulted ultimatively in a true infection. We did not have access to colony forming unit counts. The application of VAP definitions to ECMO patients harbors further challenges as non-specific alterations on chest X-ray are common in patients with ultra long-protective ventilation on ECMO (10). Given the limitations of relying on microbiologically confirmed infections, additional analyses were performed on confirmed and suspected infections. In terms of index test measurements, while the institutional protocol during the study included CRP, PCT, and WCC in addition to blood cultures to be performed regularly, there was no protocol in place to define timing on the commencement or cessation of antibiotics at the institution, nor to define suspected infection prospectively. While WCC and CRP were performed almost daily, we identified fewer PCT values, which reduced the power of the study to analyze associations with PCT. Finally, we did not investigate immature to total ratio.

Our findings indicate that current infection markers are neither sensitive nor specific enough to reliably diagnose infection in neonates and children on ECMO. Difficulty in diagnosis combined with altered pharmacokinetics of medications on ECMO makes early effective treatment challenging. New biomarkers, such as host gene expression-based signatures, have been suggested to improve the guidance on antibiotic treatment decisions (44, 45). These markers have great potential to improve ECMO outcomes by facilitating earlier diagnosis of infection. Future research should develop and validate prediction models that estimate the risk of having a bacterial or fungal infection, based on all the available information including clinical signs and new infection markers. Implementation studies of these prediction models should then test whether the use of the models helps reduce unnecessary antibiotic exposure, resulting in improved antimicrobial stewardship at non-inferior safety.

In conclusion, while CRP performed best to diagnose infection for children on ECMO, its diagnostic accuracy was only moderate. Increases in WCC, and combinations of CRP and WCC or PCT, although commonly used in practice, did not result in improved diagnostic performance. Larger prospective studies are needed to test novel markers of infection to improve recognition of infection in patients on ECMO.

## Data Availability Statement

The datasets presented in this article are not readily available because requests for additional analyses on the data need clearance from the institutional ethics board. Requests to access the datasets should be directed to the corresponding author.

## Ethics Statement

The studies involving human participants were reviewed and approved by HREC/17/QRCH/8. Written informed consent to participate in this study was provided by the participants' legal guardian/next of kin.

## Author Contributions

LJS designed the study, supervised analyses, interpreted the data, and wrote the first draft and the final manuscript. ID contributed to study design, collected all the data, performed initial analyses, and wrote with LJS the first draft. LPS and AS performed statistical analyses and contributed to manuscript writing. AM, EH, and NA were involved in study design, data interpretation, and manuscript writing. All authors read and approved the final manuscript.

## Funding

LJS was supported by a Practitioner Fellowship of the National Health and Medical Research Council of Australia and New Zealand, and by the Children‘s Hospital Foundation, Brisbane, Australia. This study was performed at the Queensland Children's Hospital, Brisbane, Australia.

## Conflict of Interest

The authors declare that the research was conducted in the absence of any commercial or financial relationships that could be construed as a potential conflict of interest.

## Publisher's Note

All claims expressed in this article are solely those of the authors and do not necessarily represent those of their affiliated organizations, or those of the publisher, the editors and the reviewers. Any product that may be evaluated in this article, or claim that may be made by its manufacturer, is not guaranteed or endorsed by the publisher.

## Abbreviations

ARDS: acute respiratory distress syndrome
BSI: blood stream infection
CLABSI: central line associated blood stream infection
ECMO: extracorporeal membrane oxygenation
ECPR: extracorporeal cardiopulmonary resuscitation
SIRS: systemic inflammatory response syndrome
VA ECMO: venoarterial ECMO
VAP: ventilator associated pneumonia
VV ECMO: venovenous ECMO.
