# Supplementary material for: Diagnostic Accuracy of Infection Markers to Diagnose Infections in Neonates and Children Receiving Extracorporeal Membrane Oxygenation
Source: Front Pediatr. 2022 Jan 26;9:824552. doi: 10.3389/fped.2021.824552 (PMC8826436; doi:10.3389/fped.2021.824552)
Supplement: Supplementary file 1 [file Data_Sheet_1.docx]

***Supplementary Material:***

**Diagnostic Accuracy of Infection Markers to Diagnose Infections in Neonates and Children Receiving Extracorporeal Membrane Oxygenation**

Irene Doo MBBS B.Sc^1,2^, Lukas P Staub^3^, PhD, Adrian Mattke^,1,2^, FCICM, Emma Haisz^1,2^, MBBS, Anna Lene Seidler^3^, MSc, Nelson Alphonso^4^, FRACS, Luregn J Schlapbach MD, PhD, FCICM ^1,2,5^

**Supplementary Figure 1: Diagnostic accuracy of White Cell Count to diagnose infection in neonates and children on ECMO.**

**Supplementary Figure 2: Optimal cut-offs of C-Reactive Protein and Procalcitonin to diagnose infection in neonates and children on ECMO.**

**Supplementary Figure 3: Time course of White Cell Count, C-Reactive Protein and Procalcitonin to diagnose infection in neonates and children on ECMO.**

**Supplementary Table A: Diagnostic procedures performed, focus of infection and pathogens identified in 65 neonatal and pediatric ECMO runs.**

**Supplementary Table B. Association between infection markers and ECMO parameters.**

**Supplementary Figure 1: Diagnostic accuracy of White Cell Count to diagnose infection in neonates and children on ECMO.** Graphs show the Receiver-Operating Characteristic (ROC) Area Under the Curve (AUC) for confirmed infection (A), and for confirmed and suspected infection (B). Dark grey shaded areas indicate 95%-Confidence bands.

1. ***Confirmed infections B) Suspected and confirmed infections***


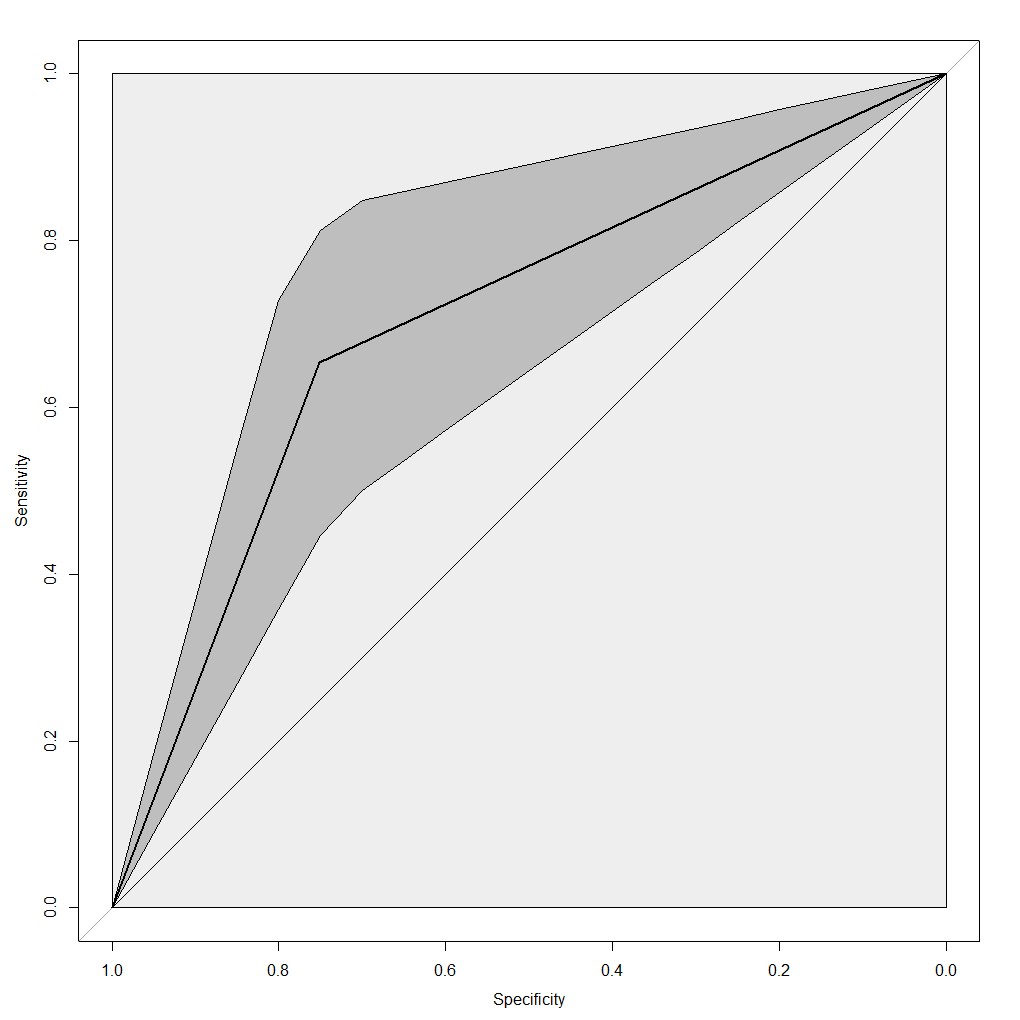
**
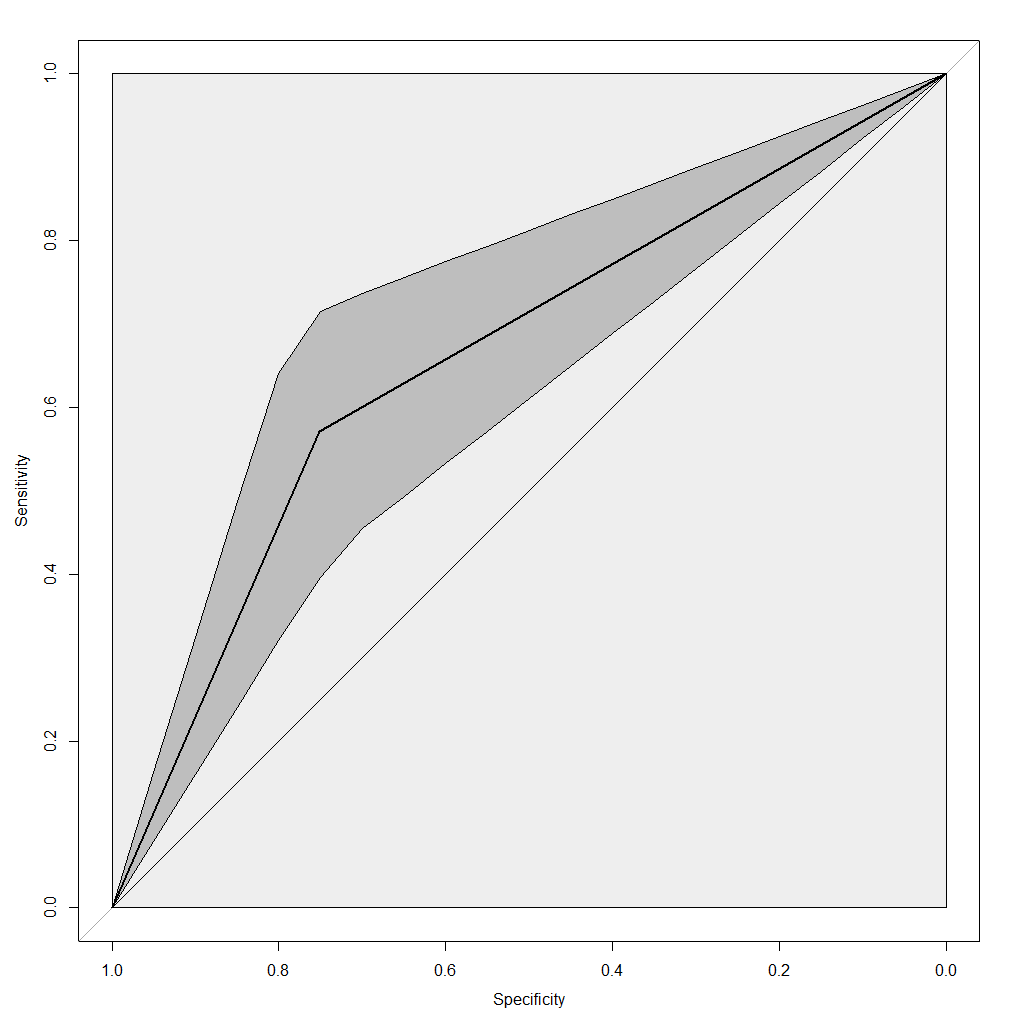
**

**Supplementary Figure 2: Optimal cut-offs of C-Reactive Protein (CRP; panel A) and Procalcitonin (PCT, panel B) to diagnose infection in neonates and children on ECMO.** Graphs show the sensitivity achieved for each biomarker threshold in children with infection versus controls. Intersections are set at 80% and 90% sensitivity, respectively.

1. ***CRP***


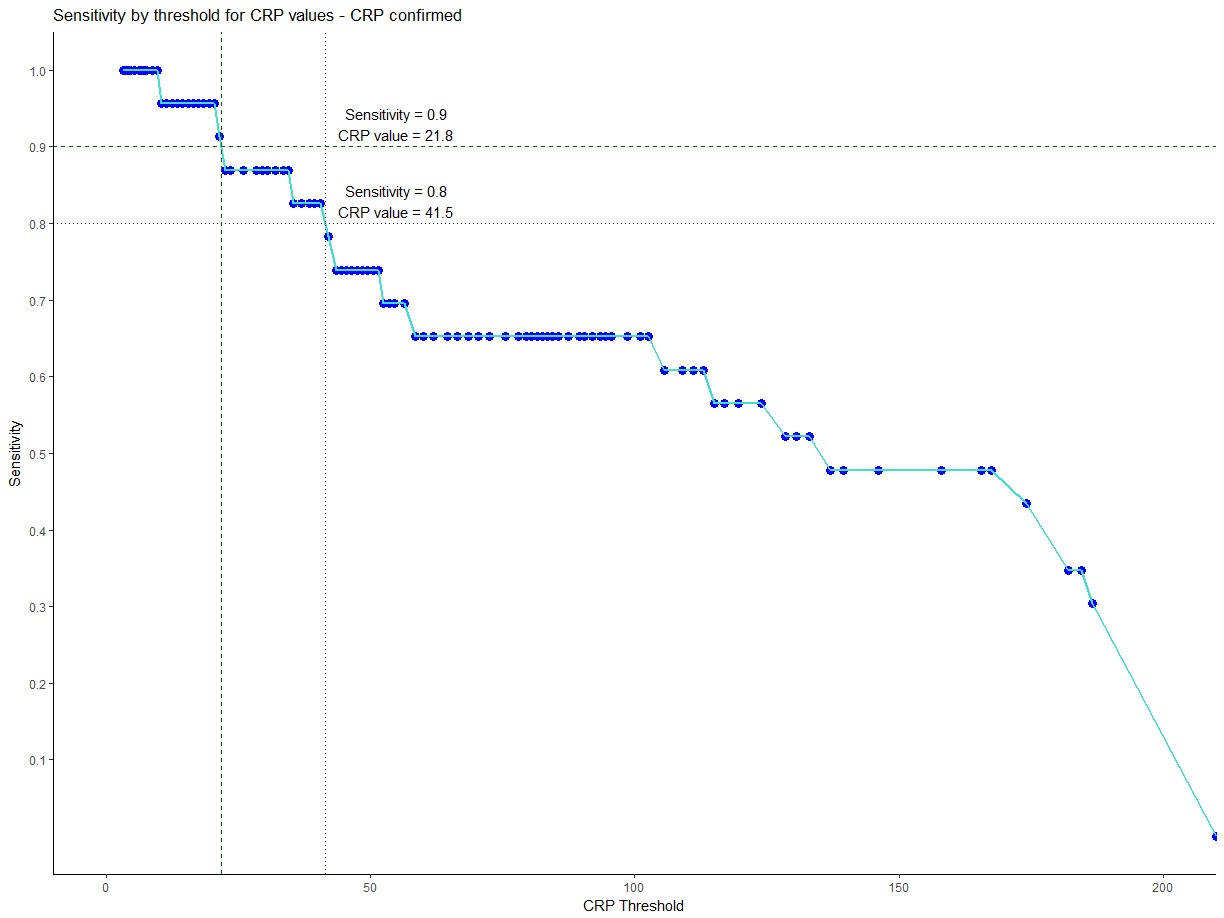


1. ***PCT***

**
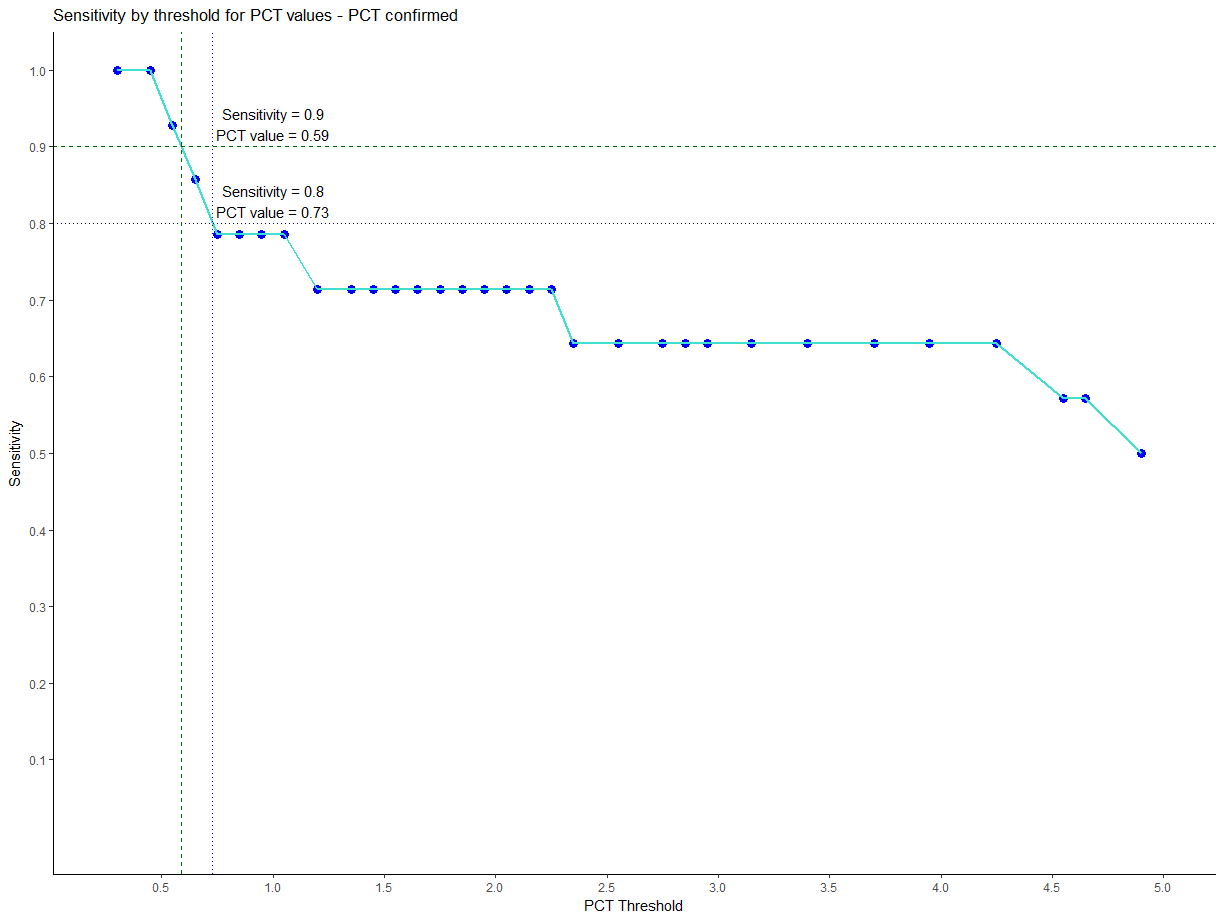
**

**Supplementary Figure 3: Time course of White Cell Count (WCC, Panel A and B), C-Reactive Protein (CRP; panel C and D) and Procalcitonin (PCT, panel E and F) to diagnose infection in neonates and children on ECMO.** Graphs show the time course of marker levels in relation to day after onset of infection (A, C, E), and in relation to day of ECMO (B, D, F). Estimates (blue line) and 95% confidence intervals (grey shaded area) are shown.

1. ***WCC by ECMO day after onset of infection.***


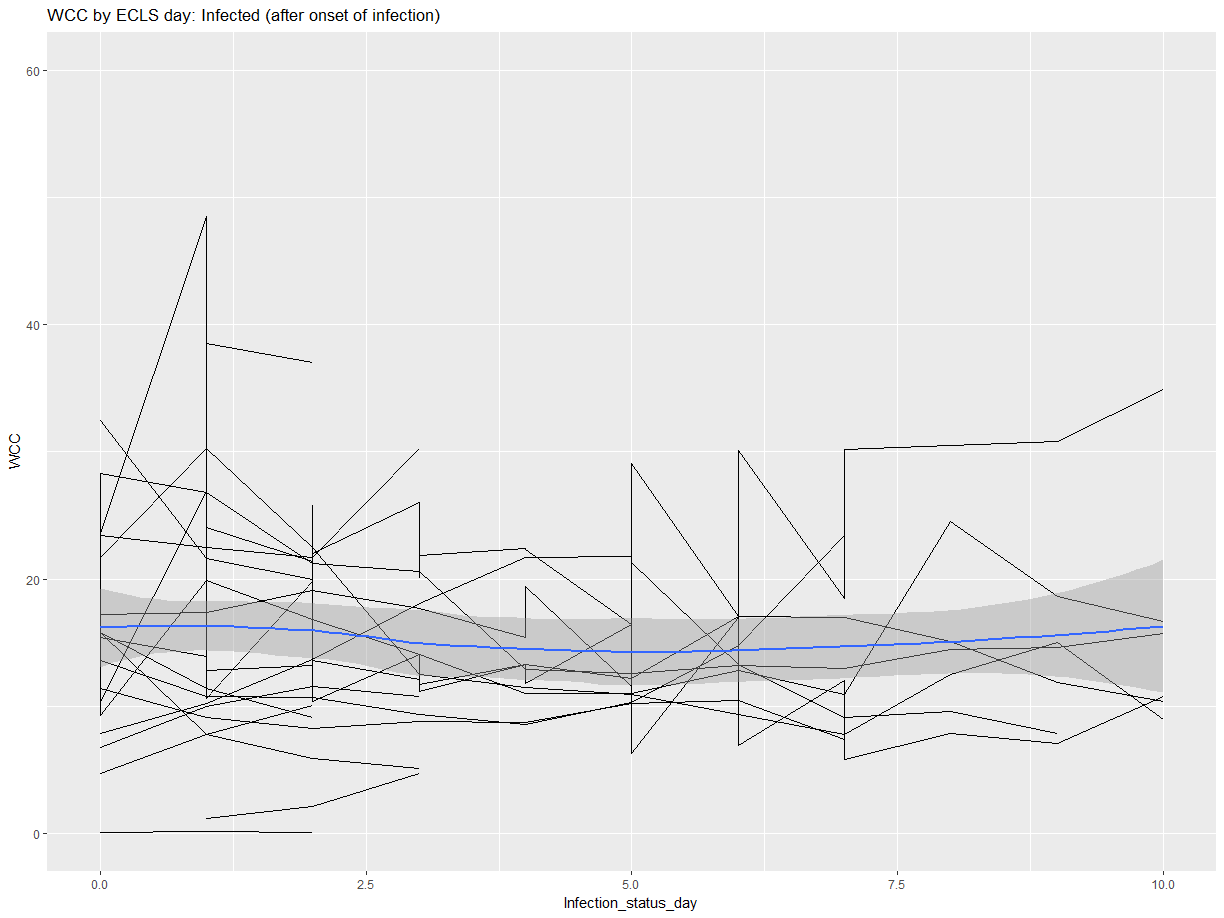


1. ***WCC by ECMO day***


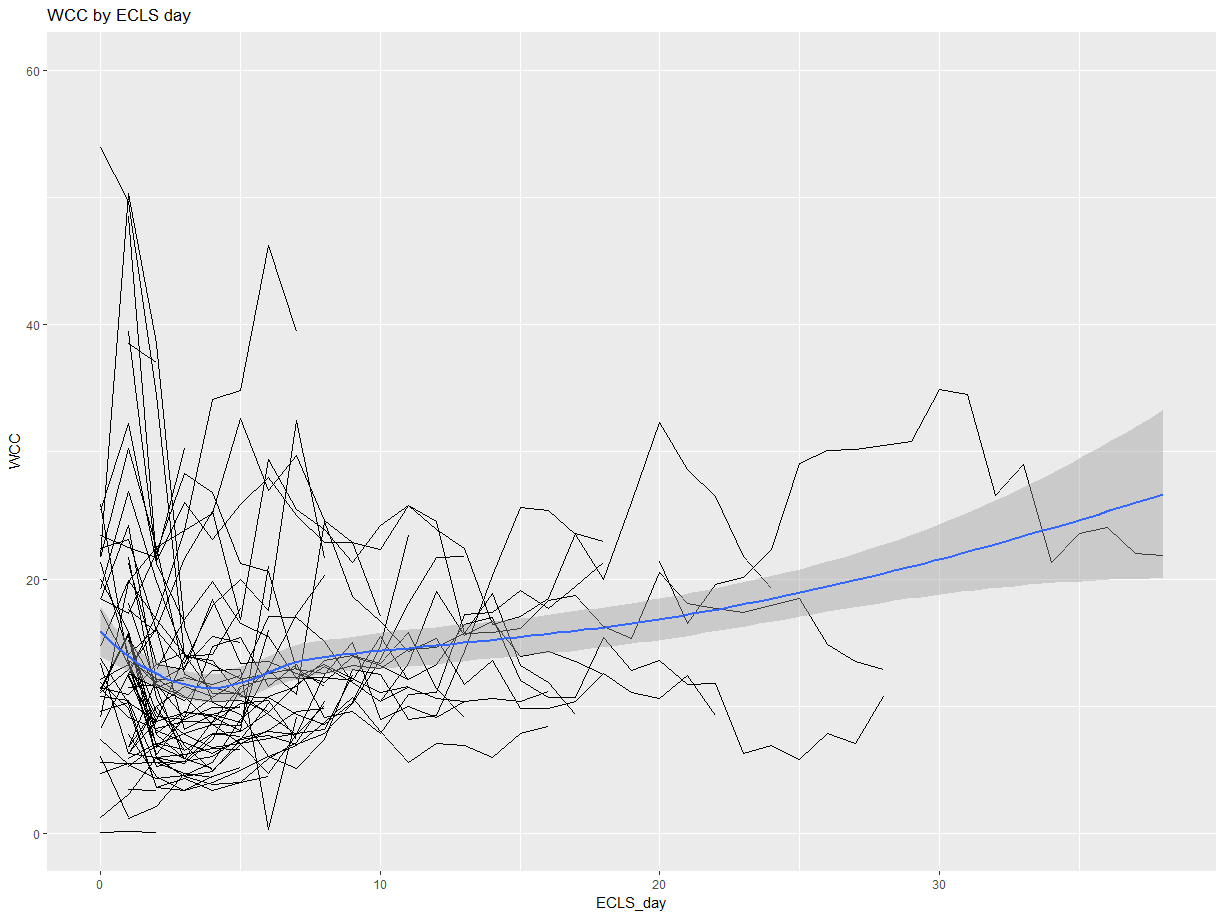


1. ***CRP by ECMO day after onset of infection.***

***
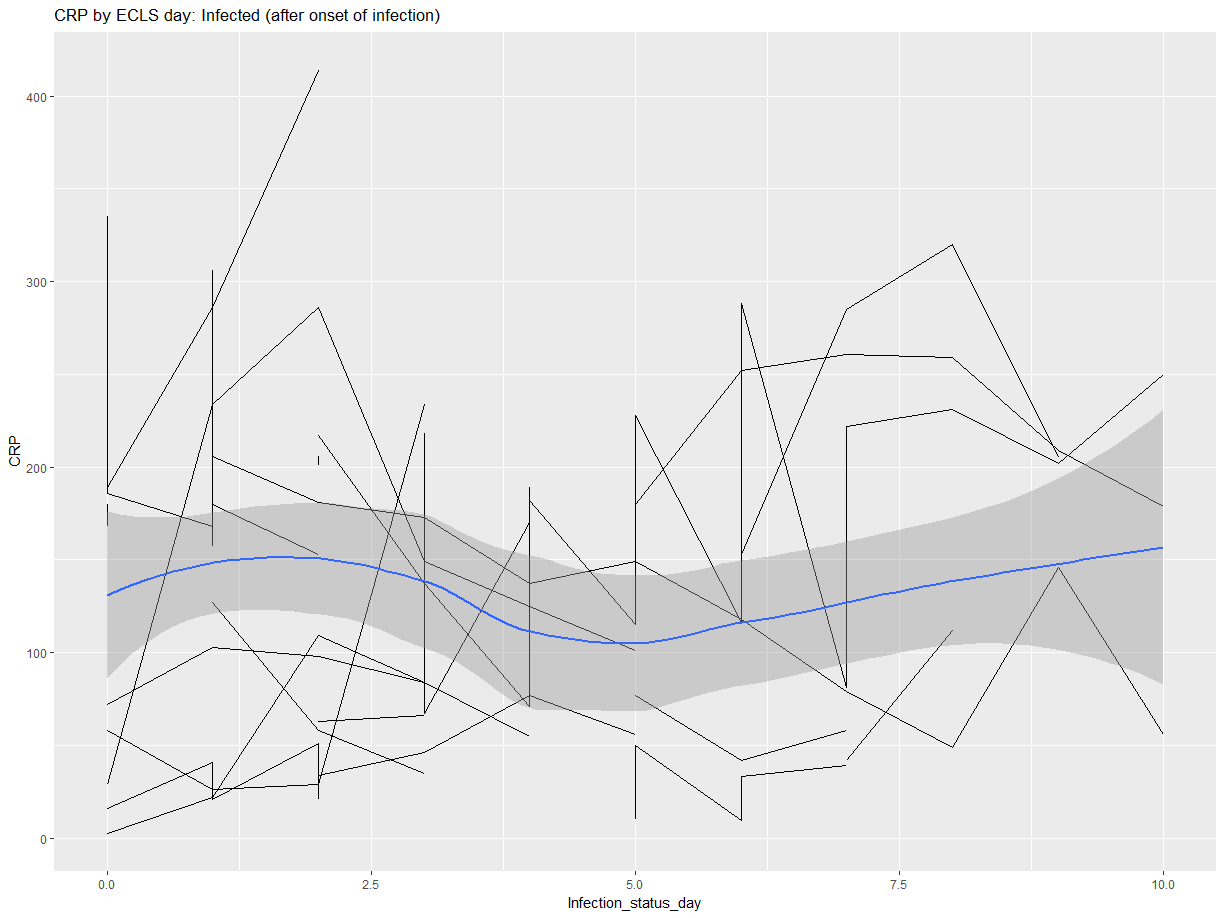
***

1. ***CRP by ECMO day***

***
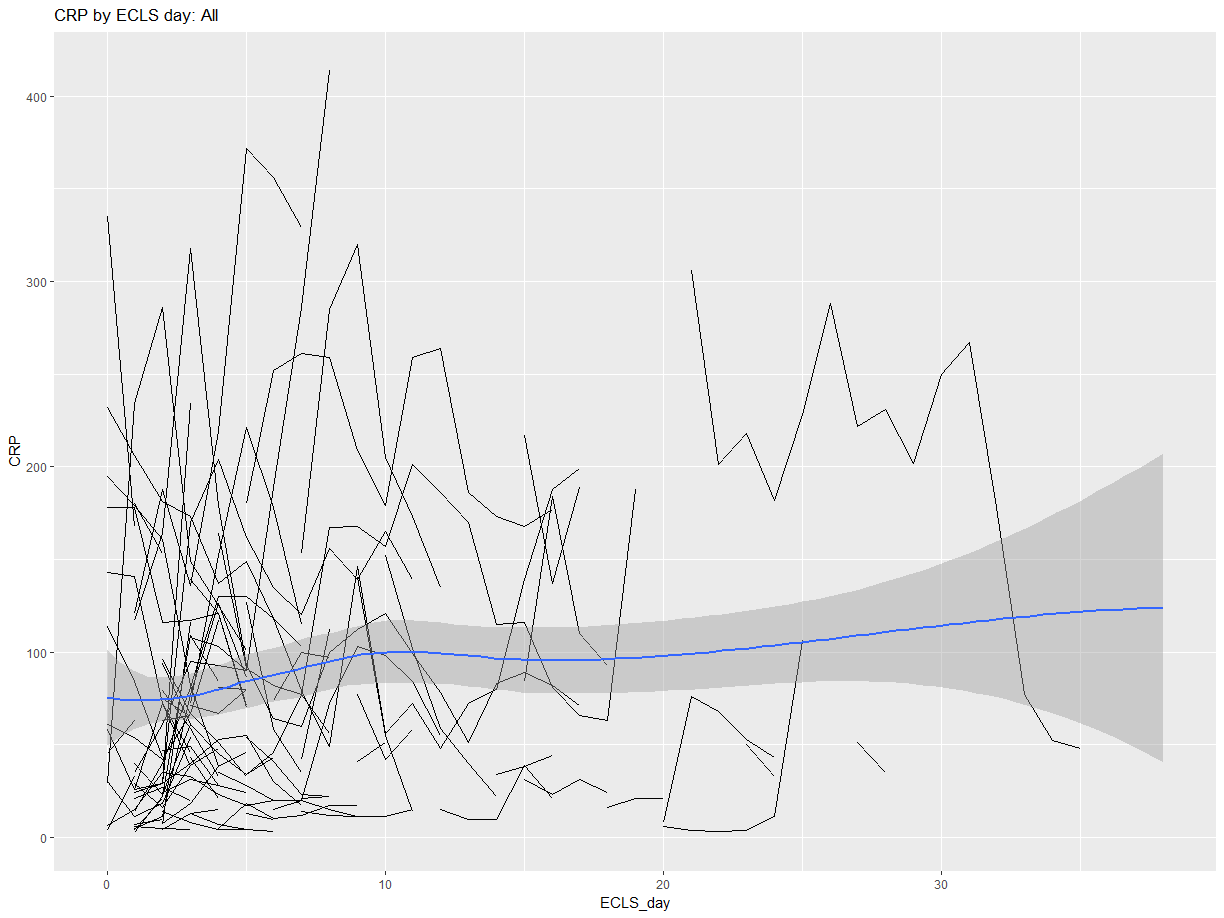
***

1. ***PCT by ECMO day after onset of infection.***

***
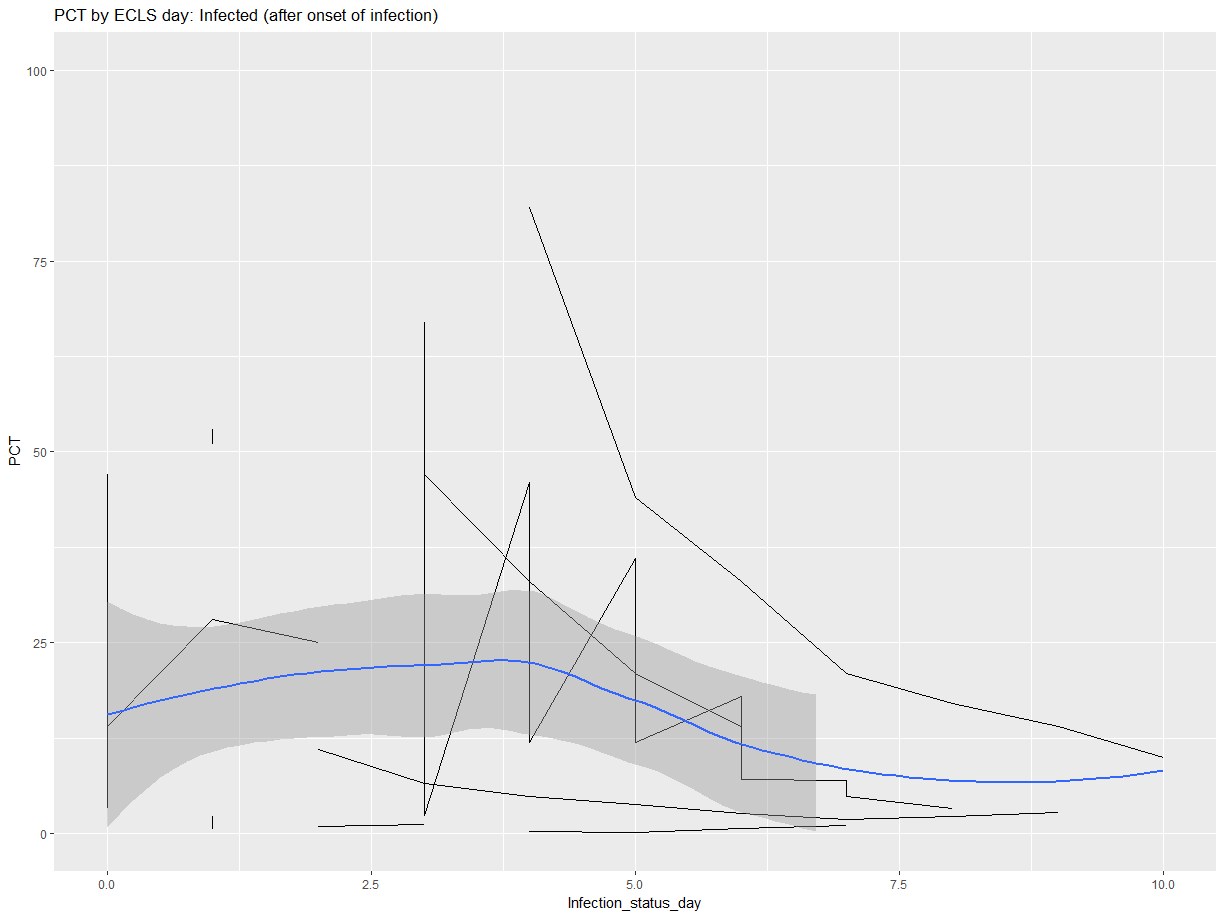
***

1. ***PCT by ECMO day***


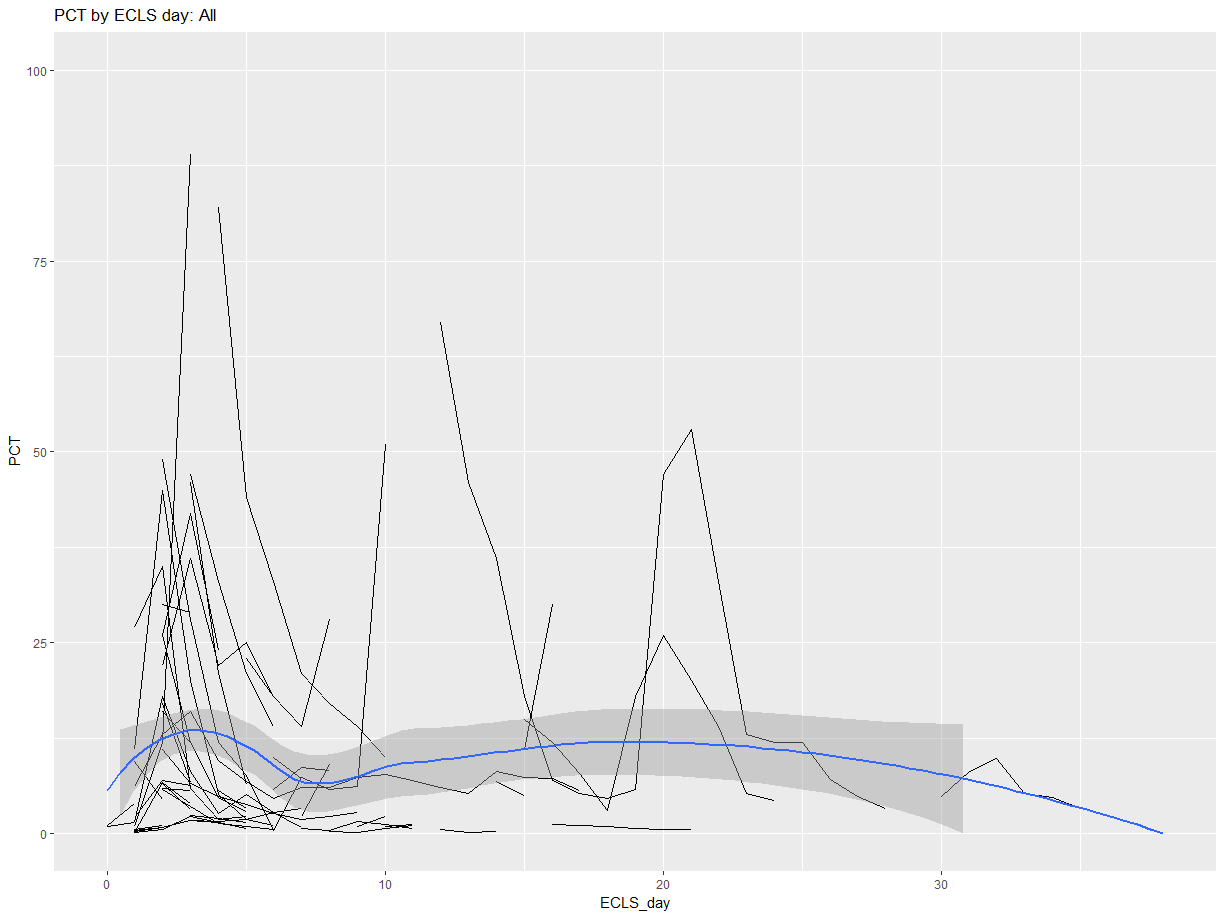


**Supplementary Table A: Diagnostic procedures performed, focus of infection and pathogens identified in 65 neonatal and pediatric ECMO runs.** Counts represent days with the respective diagnostic test performed, focus of infection, and days with a culture that resulted positive. Please note that some patients had multiple positive cultures.

|  | **Runs with no infection** | **Runs with suspected infection** | **Runs with confirmed infection** |
| --- | --- | --- | --- |
| **Variable** | **N (%)** | **N (%)** | **N (%)** |
| ***Total days*** | *182* | *129* | *264* |
| Blood culture | 142 (78) | 110 (85) | 240 (91) |
| Endotracheal aspirate | 27 (15) | 31 (24) | 86 (33) |
| Urine culture | 21 (12) | 22 (17) | 45 (17) |
| Nasopharyngeal aspirate | 10 (5) | 9 (7) | 25 (9) |
| C-reactive protein | 124 (68) | 94 (73) | 186 (70) |
| Procalcitonin | 84 (46) | 67 (52) | 101 (38) |
| White Cell Count | 175 (96) | 125 (97) | 257 (97) |
| ***Focus of infection (days)*** |  |  |  |
| unknown | NA | 24 | 2 |
| Bloodstream infection | NA | 0 | 38 |
| Ventilator-associated pneumoniae | NA | 34 | 153 |
| BSI and VAP | NA | 0 | 3 |
| Urinary tract infection | NA | 0 | 0 |
| VAP and UTI | NA | 0 | 13 |
| Would infection | NA | 0 | 0 |
| Abdominal infection | NA | 0 | 0 |
| Other infection | NA | 0 | 15 |
| ***Identified pathogens*** |  |  |  |
| **Blood culture** |  |  |  |
| *S. aureus* |  |  | 5 |
| *CoNS* |  |  | 4 |
| *S. pneumoniae* |  |  | 2 |
| *E.coli* |  |  | 2 |
| *Stenotrophomonas* |  |  | 3 |
| *Acinetobacter* |  |  | 1 |
| *Candida spp.* |  |  | 1 |
| **Endotracheal culture** |  |  |  |
| *S. aureus* |  |  | 1 |
| *CoNS* |  |  | 6 |
| *Streptococcus* |  |  | 4 |
| *Enterococcus* |  |  | 3 |
| *Enterobacter* |  |  | 7 |
| *Pseudomonas* |  |  | 3 |
| *Klebsiella spec* |  |  | 7 |
| *Serratia* |  |  | 2 |
| *Stenotrophomonas* |  |  | 13 |
| other bacteria |  |  | 2 |
| *Candida spp.* |  |  | 13 |
| **Urine culture** |  |  |  |
| *E. coli* |  |  | 1 |
| *Enterobacter* |  |  | 1 |
| *Pseudomonas* |  |  | 3 |
| *Candida spp.* |  |  | 13 |

CoNS, Coagulase negative staphylococci; BSI, bloodstream infection; VAP, ventilator-associated pneumonia; UTI, urinary tract infection; NA, not applicable.

**Supplementary Table B. Associations between infection markers and ECMO parameters.** Comparison of estimates (and 95% confidence intervals) of infection markers in the ECMO type groups and in two age groups; trends in infection markers over time; and association of infection markers with increasing peak daily lactate and daily flow rate.

|  | **PCT [mg/L]** | **CRP [mg/L]** | **WCC [10^9^/L]** |
| --- | --- | --- | --- |
| **ECMO type** |  |  |  |
| VA (n=467) | 33.25 (8.34 to 58.17) | 70.42 (53.63 to 87.18) | 12.33 (9.33 to 15.32) |
| VV (n=122) | 7.3 (-54.83 to 69.40) | 76.34 (38.64 to 114.08) | 20.15 (13.33 to 26.99) |
|  |  |  |  |
| **Patient age*** |  |  |  |
| Pediatric (n=361) | 43.72 (13.69 to 73.83) | 90.79 (71.79 to 109.62) | 16.66 (13.08 to 20.24) |
| Neonatal (n=237) | 10.19 (-34.68 to 54.97) | 50.48 (21.82 to 79.27) | 10.27 (4.95 to 15.59) |
|  |  |  |  |
| **ECMO day** |  |  |  |
| Intercept (day 0) | 32.28** | 72.65 | 0.10** |
| Change per day | -0.73 (-1.28 – -0.18)** | 0.1 (-0.84 to 1.05) | -0.12 (-0.22 to -0.02)** |
|  |  |  |  |
| **Peak daily lactate** |  |  |  |
| Change in marker per unit change of lactate | -0.28 (-1.31 to 0.77) | -2.13 (-4.58 to 0.34) | 0.46 (0.23 to 0.7)** |
|  |  |  |  |
| **Peak daily flow rate** |  |  |  |
| Change in marker per unit change of flow rate | 12.01 (5.31 to 18.74)** | 21.66 (6.33 to 37.22)** | -0.57 (-2.26 to 1.12) |

* age cut-off: 4 weeks

** statistically significant p<0.05
